# Transcription Profiling Analysis of Mango–*Fusarium mangiferae* Interaction

**DOI:** 10.3389/fmicb.2016.01443

**Published:** 2016-09-14

**Authors:** Feng Liu, Jing-bo Wu, Ru-lin Zhan, Xiong-chang Ou

**Affiliations:** Key Laboratory of Tropical Fruit Biology, Ministry of Agriculture, Southern Subtropical Crops Research Institute, Chinese Academy of Tropical Agricultural SciencesZhanjiang, China

**Keywords:** mango, mango malformation disease, transcriptome, gene expression, plant–pathogen interaction

## Abstract

Malformation caused by *Fusarium mangiferae* is one of the most destructive mango diseases affecting the canopy and floral development, leading to dramatic reduction in fruit yield. To further understand the mechanism of interaction between mango and *F. mangiferae*, we monitored the transcriptome profiles of buds from susceptible mango plants, which were challenged with *F. mangiferae*. More than 99 million reads were deduced by RNA-sequencing and were assembled into 121,267 unigenes. Based on the sequence similarity searches, 61,706 unigenes were identified, of which 21,273 and 50,410 were assigned to gene ontology categories and clusters of orthologous groups, respectively, and 33,243 were mapped to 119 KEGG pathways. The differentially expressed genes of mango were detected, having 15,830, 26,061, and 20,146 DEGs respectively, after infection for 45, 75, and 120 days. The analysis of the comparative transcriptome suggests that basic defense mechanisms play important roles in disease resistance. The data also show the transcriptional responses of interactions between mango and the pathogen and more drastic changes in the host transcriptome in response to the pathogen. These results could be used to develop new methods to broaden the resistance of mango to malformation, including the over-expression of key mango genes.

## Introduction

The fruit of mango (*Mangifera indica* L.) is exceptional for its spicy, succulent, thick fruit pulp and abundant nutrients, containing a wide range of amino acids, sugar, organic acids, and minerals including Ca, P, Fe, and K as well as a great variety of vitamins. Mango cultivation is affected by specific constraints, among which mango malformation disease (MMD) caused by *Fusarium mangiferae* is considered to be one of the most important threats in the majority of mango-planting regions worldwide (Ploetz et al., 2002; Marasas et al., 2006; Zhan et al., 2010, 2012). In the past decade, MMD has destroyed many thousands of hectares of mango in tropical and subtropical countries. Because of the economic importance of MMD, many studies have been performed on the occurrence (Steenkamp et al., 2000; Lima et al., 2009; Iqbal et al., 2011b), pathogen genetic diversity (Iqbal et al., 2006; Liu et al., 2014), pathogen detection (Wu et al., 2016), pathogen cytology (Iqbal et al., 2010), infection life cycle (Freeman et al., 1999; Gamliel-Atinsky et al., 2009), and chemical control (Iqbal et al., 2011a) of the disease, but research on the screening of mango germplasm for MMD resistance and on molecular mechanisms underlying MMD resistance and the pathogenicity of *F. mangiferae* is scarce (Singh, 2006). The main control measures against MMD include destruction of diseased mango branches, use of disease-free plant materials and fungicides. However, no fungicides or other chemical means have been proven effective for the control of this disease (Iqbal et al., 2011b). Although cultural practices such as heavy pruning can influence the development of the disease, none can efficiently control MMD. Thus, breeding for mango cultivars with durable resistance to MMD is considered to be one of the most economical, environmentally safe, and effective strategies for disease management. Although no mango variety has yet been identified as having complete, or even a high level of, resistance, cultivars do exhibit significant differences in quantitative resistance to *F. mangiferae*. However, a lack of genetics resources within conventional breeding programs demonstrated that attempts to improve tolerance to MMD have not been successful.

A multi-layered process occurs between plants and pathogens, and deciphering the molecular basis of the interactions would significantly assist the development of new control strategies. In the past, efforts have been made to discover the molecular mechanisms underlying interactions between plants and pathogens (Boyd et al., 2013; Orlowska et al., 2013). Plants have evolved multiple strategies to defend against damage from various attackers, such as pathogens, insects, and fungi (Dang and Jones, 2001; Wise et al., 2007). These strategies involve two complex mechanisms of interaction, namely, PAMP-triggered immunity (PTI), and effector-triggered immunity (ETI; Bonas and Lahaye, 2002; Göhre and Robatzek, 2008), and three main plant defense hormones: salicylic acid (SA), jasmonic acid (JA), and ethylene (ET; Sato et al., 2010; Miljkovic et al., 2012). PTI is the earliest response of plants under attack, when host receptors recognize the pathogen-derived PAMP (pathogen-associated molecular pattern), whereas ETI is prompted by the interaction between a pathogenic effector and a “Resistance” protein (McDowell and Simon, 2008; Zhang and Zhou, 2010). The two immune systems result in a non-host resistance (considered as PTI) and a partial or qualitative resistance (considered as ETI). JA and ET are generally involved in the defense against necrotrophic pathogens and herbivorous insects, whereas SA is involved in immunity against biotrophic and hemibiotrophic pathogens (Dong, 1998). The availability of modern molecular biological techniques, such as RNA sequencing, provides the new insights into molecular mechanisms of plant resistance during the interaction of plants and pathogens. This technique has been performed for many host–pathogen interactions, including banana and *F. oxysporum* f. sp. *cubense* (Li et al., 2013), tomato and *Xanthomonas perforans* race T3 (Du et al., 2015), oil palm and *Ganoderma* spp. (Ho et al., 2016), pea and *Phytophthora pisi* (Hosseini et al., 2015), wheat and *Heterodera avenae* (Kong et al., 2015), and soybean and *F. oxysporum* (Lanubile et al., 2015). Many genes were revealed to be involved in defense mechanism and resistance-associated signal transduction in plants. For instance, phytohormone-related genes were found to be significantly up-regulated in potato after *Ralstonia solanacearum* inoculation (Zuluag et al., 2015). MMD is an emerging fungal disease that causes severe yield loss in mango. However, little is known about the molecular mechanisms underlying the interactions between mango and *F. mangiferae*. This study was carried out to further a better understanding of the interactions between mango and the pathogen in order to develop effective means to control this important mango disease.

## Materials and methods

### Plant material and pathogen inoculation

The higly virulent strain of *F. mangiferae* (MG06) isolated from an infected Keitt mango (Zhan et al., 2012) and preserved in the lab of SSCRI was used in this study. The pahtogen was inoculated into 1-year-old “Keitt” mango seedlings planted in 10^−L^ (plastic) pots in a glasshouse. The inoculum was in the form of conidial suspension obtained by adding (10 mL) sterile water to a 7 day-old 9-cm PDA culture plate to dislodge the conidia, and followed by filtering the suspension through two layers of sterile cheesecloth. Wound inoculation was performed by injecting 200 μL of conidial suspension (1 × 10^6^ spores per mL) into the axillary or apical buds. Water-inoculated plants served as the control. All plantlets were kept in a greenhouse at 25–32°C with a 16-h photoperiod. Fifteen buds were harvested at 45, 75, and 120 days post-inoculation (dpi). The control was an equal mixture of buds harvested from the water-inoculated samples at the same time intervals. The treated tissues were quickly frozen in liquid nitrogen and stored at −80°C. The samples were marked as CK (control), 45D, 75D, and 120D.

### Extraction and purification of total RNA

Frozen buds were ground mechanically into fine powder in liquid nitrogen. Total RNA was isolated using a Quick RNA Isolation Kit (Huayueyang Biotechnology, Beijing, China), in accordance with the manufacturer's guidelines. The total RNA was resuspended in RNase-free water, and RNA integrity and quality were assessed using an Agilent 2100 Bioanalyzer (Agilent, Santa Clara, CA, USA).

### RNA processing for transcriptome sequencing

Poly (A) mRNA was isolated using oligo-dT beads (Qiagen). All mRNAs were broken into short fragments by adding a fragmentation buffer. First-strand cDNA was generated using random hexamer-primed reverse transcription, followed by synthesis of the second-strand cDNA by using RNase H and DNA polymerase I. The cDNA fragments were purified using a QIAquick PCR extraction kit. These purified fragments were then washed with EB buffer for end reparation poly (A) addition and ligated to sequencing adapters. Following agarose gel electrophoresis and extraction of cDNA from gels, the cDNA fragments were purified and enriched by PCR to construct the final cDNA library. The cDNA library was sequenced on the Illumina sequencing platform (Illumina HiSeq™ 2500) by using the paired-end technology from Gene Denovo Co. (Guangzhou, China).

### *De novo* assembly and annotation

A Perl program was written to select clean reads by removing low-quality sequences (those in which more than 50% of bases presented quality of ≤10), reads with more than 5% N bases (bases unknown), and reads containing adaptor sequences. Clean reads were *de novo* assembled by Trinity Program (Grabherr et al., 2011). All the unigenes were then compared with four protein databases, namely, NCBI non-redundant protein database (Nr; http://www.ncbi.nlm.nih.gov/), Clusters of Orthologous Groups of proteins database (COG; http://www.ncbi.nlm.nih.gov/COG/), KEGG, and Swiss-Prot (http://www.expasy.ch/sprot), by using BLASTX (Altschul et al., 1997) with an *E*-value cutoff of 10^−5^. The sequence direction of the unigenes was determined using the optimum alignment results. When the results were conflicted among databases, the direction was determined consecutively by Nr, Swiss-Prot, KEGG, and COG. When a unigene would not align to any database, ESTScan (http://myhits.isb-sib.ch/cgi-bin/estscan) was used to predict the coding regions and determine the sequence direction. GO annotation was analyzed by Blast2GO software (https://www.blast2go.com/). Functional classification of the unigenes was performed using WEGO software (Ye et al., 2006).

### Identification and analysis of DEGs

Screening of DEGs back to the pipeline of bioinformatics analysis determined the genes with different expression levels among the samples, followed by GO function analysis and KEGG pathway analysis (Audic and Claverie, 1997). We developed a strict algorithm to identify the DEGs between CK and each of 45D, 75D, and 120D. FDR was used to determine the *P*-value threshold in multiple tests and analyses. We evaluated the significance of differences in gene expression by using a threshold value of absolute log_2_-fold change ≥1 with FDR ≤ 0.001 and *P* ≤ 0.005. In addition, GO and KEGG pathway enrichment analyses were performed to detect the significantly enriched functional classification and biological pathways.

### Quantitative real-time PCR

As described in the method reported by Du et al. (2015), the *Ai-actin* gene of mango was used as a reference gene. Gene-specific primers were designed using the gene sequences with Primer 5.0 software, and the primer sequences as listed in Additional File 7.

## Results

### Inoculation of mango buds with *F. mangiferae* for gene expression profiling analysis

**Figure 1:**
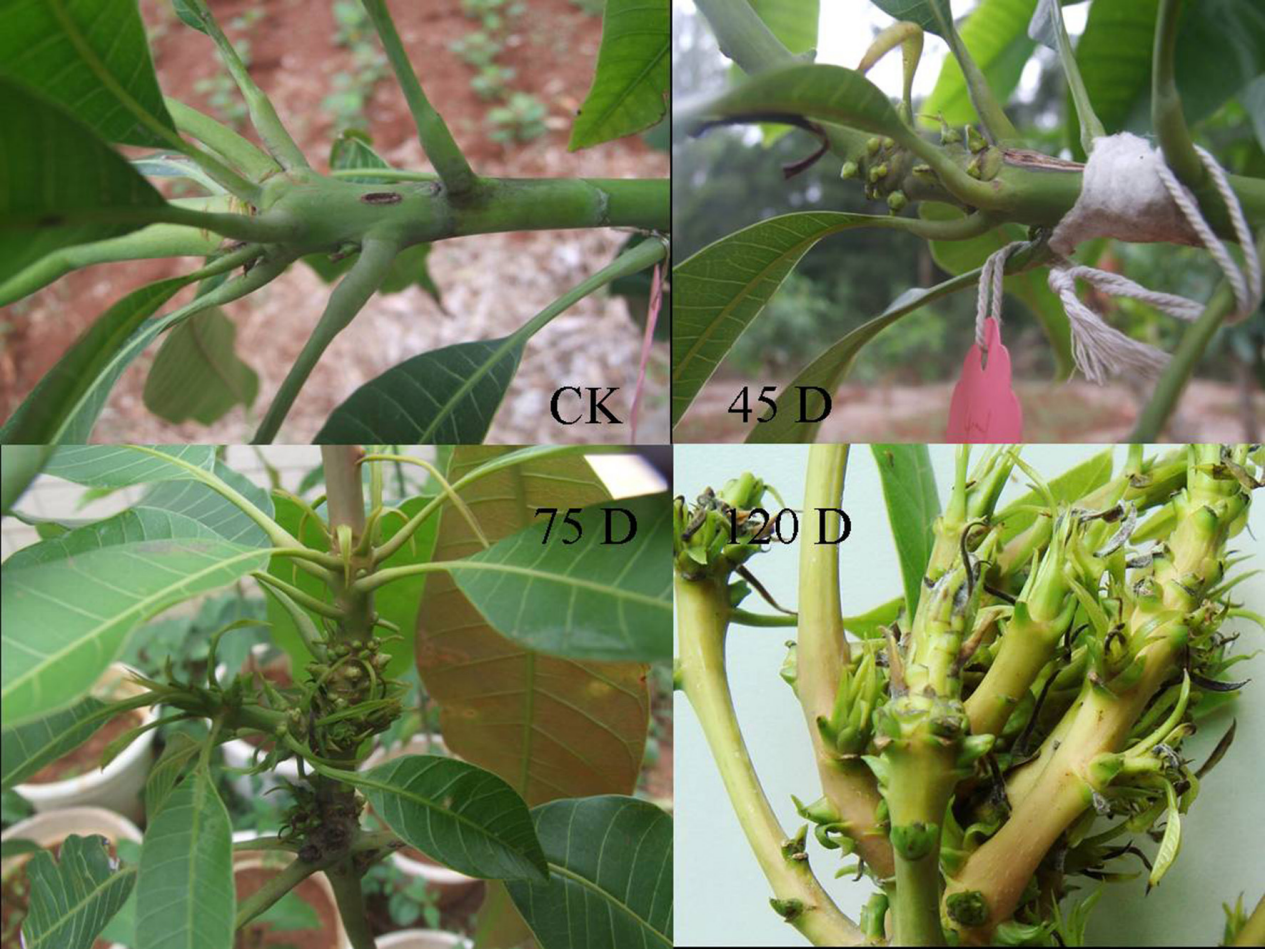
**The symptoms of mango bud after infected by ***F. mangiferae*****. CK is healthy seedling; 45D, 75D, and 120D were vegetative malformation of mango artificially after inoculated with *F. mangiferae*.

After inoculation with *F. mangiferae*, symptoms started in short internodes with the formation of a swollen bud, with small scale-like leafy structures. The growth of this shootlet was arrested, and several similar shootlets arose again from the axil of scaly leaves. This process continued. Collectively, a number of such structures gave rise to a malformed bunch (Figure 1).

To identify the genes with altered expressions in response to infection by *F. mangiferae* and to reveal any difference in global gene expression profiles at different times after inoculation, we cut the buds of mango seedlings and inoculated the wounded buds by immersing them in *F. mangiferae* spore culture. The inoculated buds were harvested at 45, 75, and 120 days after the initial inoculation for RNA extraction. The gene expression profile at the 45 day time point reflects an early host response triggered mainly by PAMPs. The profiles at 75 and 120 days are early-intermediate and late phase responses to infection by *F. mangiferae*, respectively.

### Illumina sequencing

To identify the genes with expression specifically altered when the bud is infected by *F. mangiferae*, cDNA samples were prepared from total RNA of the non-inoculated buds and buds inoculated with *F. mangiferae* for 45, 75, and 120 days. A total of 99,410,732 reads from the four libraries were subjected to *de novo* assembly (Table 1). The raw reads were deposited in the NCBI Sequence Read Archive under the accession number SRX1651783. To facilitate sequence assembly, repetitive, low-complexity, and low-quality reads were filtered out. Reliable reads were assembled into 133,077 contigs with a length from 200 to 3000 bp. After clustering the contigs together, we finally obtained 121,267 unigenes. The size was between 200 and 3000 bp, with an N50 of 1222 bp. The size distributions of these contigs and unigenes are shown in Figures S1, S2.

**Table 1:** **Output statistics of sequencing**.

**Samples**	**Total Reads**	**Total Nucleotides (nt)**	**Q20 percentage**	***N* percentage**	**GC percentage**
*Mangifera_indica*	99,410,732	9,941,073,200	98.28%	0.00%	48.27%

*Total Nucleotides = Total Reads1 × Read1 size + Total Reads2 × Read2 size; Total Reads and Total Nucleotides are actually clean reads and clean nucleotides; Q20 percentage is proportion of nucleotides with quality value larger than 20; N percentage is proportion of unknown nucleotides in clean reads; GC percentage is proportion of guanidine and cytosine nucleotides among total nucleotides*.

### Functional annotation and gene ontology classification

Functional annotation provided information on protein functions, pathways, COGs, and GO. To determine the unigenes' sequence orientation, all unigenes were aligned using BLASTX (*E* < 1 × 10^−5^) against four protein databases in the following order of priority: Nr, Swiss-Prot, KEGG, and COG. A total of 75,802 (62.51%) unigenes were successfully annotated. The largest number of annotations was found using the Nr database (60.62%), followed by Swiss-Prot (46.88%), KEGG (23.83%), and COG (19.72%; Figure S3). The remaining unaligned unigenes were analyzed using ESTscan to predict the coding regions and determine the sequence direction (Iseli et al., 1999).

Among the 121,267 unigenes, 73,509 proved to be similar to known protein sequences from *Theobroma cacao* (32.23%), *Vitis vinifera* (14.62%), *Cucumis sativus* (5.60%), *Fragaria vesca* subsp. *vesca* (5.34%), *Arabidopsis thaliana* (3.87%), *Cicer arietinum* (3.64%), *Glycine max* (3.46%), *Oryza sativa* Japonica Group (3.22%), and others (Additional File 1). Annotation of the 36,480 sequences by using the GO and COG databases yielded good results for ~65,535 unigenes and 53,816 putative proteins, respectively (Additional File 2, Figure S4, Table S1).

Table S1 shows the distributions of 53,816 unigenes assigned into 25 orthologous clusters in COG. Some unigenes may be assigned into several clusters, whereas others were assigned to the same cluster but with different protein orthologous similarities. For the majority of the unigenes (8578), only general functional predictions were possible, and the most common categories assigned were transcription (4428) and translation, as well as ribosomal structure and biogenesis (4148). A total of 2592 functionally unknown unigenes were identified, and 1617 unigenes were assigned to secondary metabolite biosynthesis, transport, and catabolism; 537 unigenes were assigned to defense mechanisms.

Figure S4 shows that 36,481 unigenes were classified into the three following GO domains: biological process, cellular component, and molecular function. One unigene may be assigned to various GO terms.

To identify the biological pathways active in mango, we mapped the annotated unigene sequences to the reference canonical pathways in KEGG. A total of 23,915 unigenes can be annotated and were assigned to 273 KEGG pathways (Additional File 3). “Ribosome” was the most common term and contained 1716 (7.18%) unigenes, followed by “Oxidative phosphorylation” (862, 3.6%) and “Protein processing in endoplasmic reticulum” (854, 3.57%).

### Digital gene expression library sequencing

**Figure 2:**
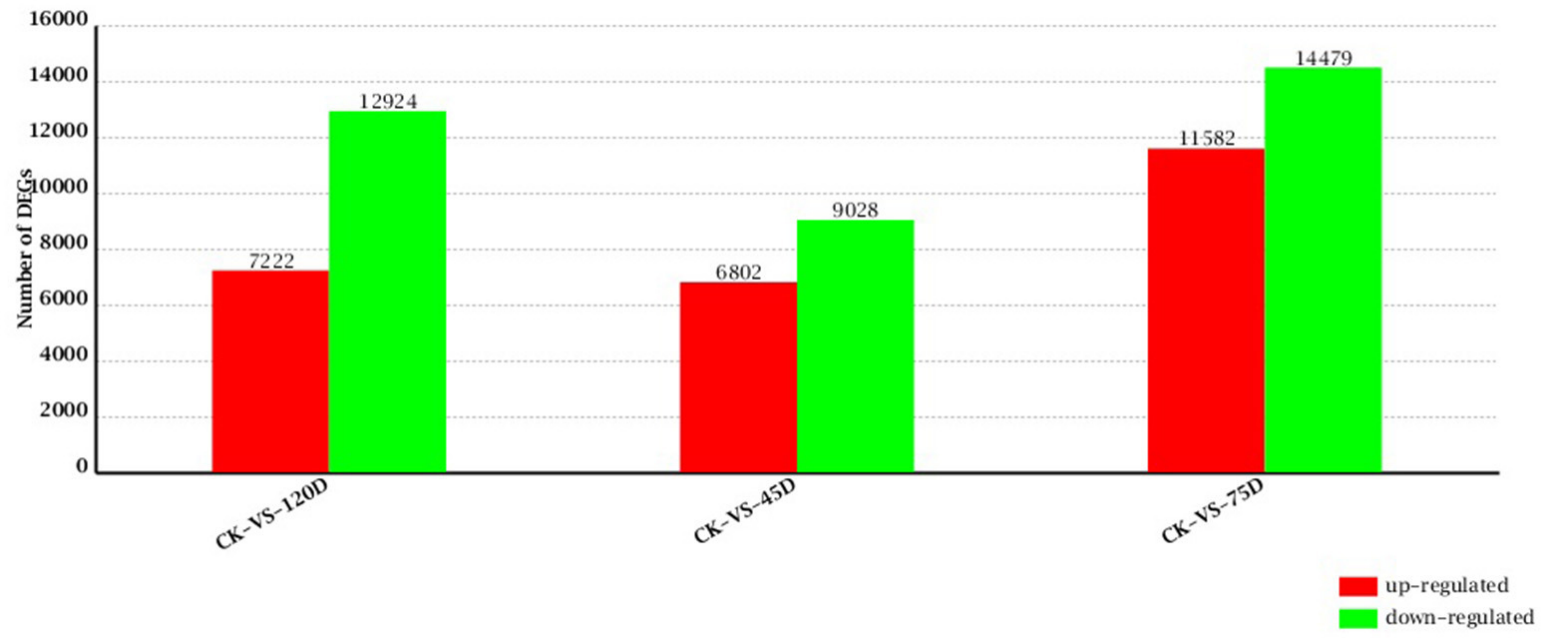
**Changes in gene expression profile of mango buds with the progression of ***F. mangiferae*** infection**. The numbers of up- and down-regulated genes in 45D, 75D, and 120D compared to CK are summarized.

A total of 15,830 genes were significantly and differentially expressed between the CK and 45D libraries, with 6802 upregulated and 9028 downregulated genes after 45 days of *F. mangiferae* inoculation. Between the CK and 75D libraries, a total of 26,061 DEGs were detected, with 11,582 upregulated and 14,479 downregulated genes (Additional File 4). There were 20,146 genes expressed at different levels in the CK and 120D libraries, with 7222 upregulated and 12,924 downregulated genes after 120 days of inoculation (Figure 2).

To focus the analysis on the genes with higher-fold changes in expression compared with the water-inoculated samples, we considered a cutoff (≥5) on the log_2_-fold change in the expression between pathogen- and water-inoculated samples of the genes with *P* ≤ 0.05. Thus, genes with more than 5-fold induction or suppression compared with water-inoculated samples were defined as DEGs.

### Gene ontology classification analysis of DEGs

To understand the biological processes associated with host reaction to *F. mangiferae* infection, GO analysis was applied to the above DEGs, and enrichment analysis was performed using an FDR-adjusted value of ≤0.05 as the cutoff. After discarding genes with unassigned function, mango DEGs were assigned to different categories. GO enrichment categorized 38.4, 35.7, and 38.2% DEGs into functional groups from the CK-VS-45D, CK-VS-75D, and CK-VS-120D comparisons, respectively. More DEGs were assigned to the terms of in the biological process and molecular function domains than to cellular component terms (Additional File 5). The dominant terms in each domain were metabolic process, cell, and catalytic activity, respectively. Although these enriched terms were similar at the different times after inoculation, the individual genes contributing to the common enriched terms were substantially diversified at different times after *F. mangiferae* inoculation.

The most significantly enriched GO terms in the biological process domain in the three inoculated libraries included “transcription, DNA-dependent” (GO: 0006351), “protein modification process” (GO: 0006464), “gene expression” (GO: 0010467), “phosphate-containing compound metabolic process” (GO: 0006796), “developmental process involved in reproduction” (GO: 0003006), “single-organism cellular process” (GO: 0044763), “response to stress” (GO: 0006950), “primary metabolic process” (GO: 0044238), and “response to hormone stimulus” (GO: 0009725; Additional File 5).

### Identification of metabolic pathways by KEGG analysis of DEGs

To further understand the functions of DEGs, we mapped them to KEGG terms to discover those genes involved in biosynthetic or signal transduction pathways that were significantly enriched. In CK-VS-45D, CK-VS-75D, and CK-VS-120D comparisons, 4177, 6640, and 5228 DEGs were mapped to 121, 124, and 122 KEGG pathways, correspondingly. Only significant pathway categories among the three comparisons were selected (Table 2). Some defense-associated biosynthetic pathways, including flavonoid biosynthesis, phenylpropanoid biosynthesis, taurine and hypotaurine metabolism, plant hormone signal transduction, zeatin biosynthesis, and ascorbate and aldarate metabolism, were significantly enriched. Four metabolic pathways, which were significant pathway categories with *P*-value of ≤0.05 at all three time points, were selected for further analysis. The selected pathways were “Flavonoid biosynthesis” (ko00941), “Phenylpropanoid biosynthesis” (ko00940), “RNA transport” (ko03013), and “Zeatin biosynthesis” (ko00908; Additional File 6).

**Table 2:** **Significantly enriched KEGG pathways of DEGs by ***F. mangiferae*** infection**.

**Pathway**	**No. of up-regulated genes**	**No. of down-regulated genes**	***p*-value**	**Pathway ID**
**CK-VS-45D**				
Flavonoid biosynthesis	28	2	8.9E−07	ko00941
Circadian rhythm—mammal	3	19	0.0011	ko04710
Ubiquitin mediated proteolysis	21	90	0.0017	ko04120
Photosynthesis	27	20	0.0018	ko00195
Phenylpropanoid biosynthesis	58	9	0.0036	ko00940
Taurine and hypotaurine metabolism	6	9	0.0036	ko00430
Stilbenoid, diarylheptanoid and gingerol biosynthesis	14	0	0.0096	ko00945
Starch and sucrose metabolism	44	53	0.0169	ko00500
Plant hormone signal transduction	67	71	0.0196	ko04075
Betalain biosynthesis	2	0	0.0305	ko00965
Other glycan degradation	2	8	0.0333	ko00511
Selenocompound metabolism	9	13	0.0419	ko00450
RNA transport	41	104	0.0428	ko03013
Zeatin biosynthesis	8	5	0.0473	ko00908
Basal transcription factors	7	28	0.0478	ko03022
**CK-VS-75D**				
Ribosome	426	201	1.33E−16	ko03010
Flavonoid biosynthesis	33	3	3.50E−05	ko00941
Phenylpropanoid biosynthesis	87	19	0.0001	ko00940
Phenylalanine metabolism	66	12	0.0003	ko00360
Phosphatidylinositol signaling system	18	46	0.0038	ko04070
Zeatin biosynthesis	13	7	0.0071	ko00908
Phagosome	74	70	0.0109	ko04145
Biosynthesis of secondary metabolites			0.0152	ko01110
RNA transport	86	140	0.0199	ko03013
Stilbenoid, diarylheptanoid and gingerol biosynthesis	17	1	0.0253	ko00945
Starch and sucrose metabolism	70	78	0.0281	ko00500
Ascorbate and aldarate metabolism	40	23	0.0351	ko00053
Glycolysis/Gluconeogenesis	140	79	0.0395	ko00010
Non-homologous end-joining	1	15	0.0401	ko03450
Isoquinoline alkaloid biosynthesis	10	4	0.0411	ko00950
Biosynthesis of unsaturated fatty acids	47	17	0.0421	ko01040
Pentose and glucuronate interconversions	63	23	0.0437	ko00040
**CK-VS-120D**				
Photosynthesis—antenna proteins	20	4	0.0015	ko00196
Fatty acid biosynthesis	15	30	0.0055	ko00061
Flavonoid biosynthesis	20	5	0.0068	ko00941
Phosphatidylinositol signaling system	8	44	0.0112	ko04070
Zeatin biosynthesis	9	6	0.0141	ko00908
Phenylpropanoid biosynthesis	52	24	0.0207	ko00940
Ubiquinone and other terpenoid-quinone biosynthesis	11	17	0.0226	ko00130
RNA transport	41	138	0.0259	ko03013
RNA degradation	24	82	0.0315	ko03018
Porphyrin and chlorophyll metabolism	8	37	0.0366	ko00860
Other types of O-glycan biosynthesis	0	5	0.0467	ko00514

By using log_2_-fold change ≥5 as a cutoff in the “Flavonoid biosynthesis” (ko00941) pathway, a total of 10 DEGs were detected with nine upregulated and one downregulated: seven chalcone synthases, one cinnamate 4-hydroxylase CYP73, one flavonoid 3′5′-hydroxylase, and one leucoanthocyanidin dioxygenase. Among the genes associated with “Phenylpropanoid biosynthesis” (ko00940), 34 DEGs were detected with 25 upregulated and nine downregulated, including six alcohol dehydrogenases, four catalase-peroxidases, six cinnamyl alcohol dehydrogenases, and eight peroxidases. In the “Zeatin biosynthesis” (ko00908) pathway, a total of 12 DEGs were detected with eight upregulated and four downregulated, including five cytokinin oxidases, two adenylate isopentenyltransferases, one cytokinin biosynthetic isopentenyltransferase, one cytokinin dehydrogenase 1-like, and one cytokinin hydroxylase. Among the genes associated with the “RNA transport” (ko03013) pathway, 102 genes showed at least 5-fold difference in their transcript levels between the CK and *F. mangiferae* inoculated buds at one or more time point (Additional File 6).

### Experimental verification of DEGs

To validate the RNA-sequencing expression profiles of mango DEGs, we monitored the expression pattern of 11 candidate DEGs at the three time points post-inoculation by using qRT-PCR. These candidate DEGs included genes related to defense response in other plant species, such as chitinase, WRKY transcription factor 26, proline-rich RLK PERK10-like, thaumatin-like protein 3, and partial genes, which were involved in secondary metabolism and hormone biosynthesis pathways. Their expression showed an approximately linear correlation to the RNA-sequencing results (Additional File 7).

## Discussion

In this study, we have investigated plant defense responses in mango following infection by *F. mangiferae*. We have studied compatible interactions between mango and *F. mangiferae*, which resulted in a disease. We hypothesize that in these compatible interactions, the transcriptomic responses in mango are linked with immunity, thus representing a failed defense response. Comparison between time points reveals distinct sets of differentially regulated genes in response to *F. mangiferae*. This finding indicates that differences in disease severity lead to disparate transcriptional changes in mango. This interpretation is strengthened by the expression patterns of genes involved in pathogen perception, in which different sets of genes are specifically and differentially regulated in response to *F. mangiferae*. This result also suggests that different signaling molecules in mango are triggered by *F. mangiferae*.

### Pathogenesis-related genes

Pathogenesis-related (PR) genes have been shown to play important roles in plant defenses against pathogen infection (Sels et al., 2008). Previous studies have demonstrated that overexpression of the PR genes encoding β-1, 3-glucanases, chitinases, and thaumatin-like proteins enhances resistance to *Ustilaginoidea virens* in rice (Han et al., 2015). Consistent with our findings, PR genes in mango have been induced by diverse biotic stresses, including infection by the anthracnose *Colletotrichum gloeosporioides* (Hong et al., 2016). We compared the transcript levels between pathogen- and water-inoculated buds at 45, 75, and 120 days post-inoculation. The transcript levels of 46 PR genes were altered by *F. mangiferae* infection (Additional File 8, Table 3), including three *PR1*, one *PR2*, one *PR4*, three *PR10*, three thaumatin-like genes (family *PR5*), 17 chitinase genes (families *PR3, PR4*, and *PR8*), 16 peroxidase genes (family *PR9*), and one proteinase inhibitor gene (family *PR6*). Most of the eight PR proteins were upregulated. Two PR proteins (Unigene0014620 and Unigene0031256) were found to be upregulated by *F. mangiferae* at all three time points. In addition, five PR proteins (Unigene0078592, Unigene0031254, Unigene0006204, Unigene0016037, and Unigene0031255) were induced only at the later time points (75 and 120D). Only one PR protein (Unigene0002883) was downregulated at the later time points (75 and 120D). A thaumatin-like protein 3 (Unigene0036253) gene was strongly induced at all three time points. Fifteen chitinase genes and 15 peroxidase genes were upregulated for at least one time point. Collectively, these differentially regulated PR genes in buds might play essential roles in mango resistance against *F. mangiferae*.

**Table 3:** **Summary of selected ***F. mangiferae***-responsive pathogenesis-related protein**.

**Gene ID**	**Gene length**	**log**_2_ **ratio**			**Description**
		**45D/CK**	**75D/CK**	**120D/CK**	
Unigene0002883	453	–	−11.76	−11.76	Pathogenesis-related protein 1-like protein
Unigene0014620	780	1.51	5.04	4.04	Pathogenesis-related protein P2
Unigene0031256	522	1.89	5.53	4.40	Pathogenesis-related protein 10.5
Unigene0031254	371	–	7.44	6.68	Pathogenesis-related protein 10.5
Unigene0006204	753	–	10.46	–	Pathogenesis-related protein 1-like protein
Unigene0016037	462	–	11.16	–	Pathogenesis-related protein PR-4B
Unigene0031255	396	–	14.35	–	Pathogenesis-related protein 10.5
Unigene0081141	427	12.10	–	12.21	Pathogenesis-related protein 1-like protein
Unigene0013360	646	–	–	−11.52	Thaumatin-like protein 1-like isoform X2
Unigene0036253	1280	2.73	6.07	5.67	Thaumatin-like protein 3, partial
Unigene0084433	319	–	12.20		Thaumatin-like protein
Unigene0035953	607	−14.50	−14.50	−14.50	Chitinase
Unigene0016244	550	–	−11.41	−11.41	Chitinase
Unigene0039347	1360	1.49	5.50	3.47	Chitinase 5-like isoform 1
Unigene0036325	907	4.01	6.21	4.89	Chitinase 7
Unigene0038818	838	–	6.31	4.36	Chitinase
Unigene0042899	1031	3.06	6.38	5.99	Endochitinase CH5B-like
Unigene0088278	460	–	11.75	–	Endochitinase CH5B-like
Unigene0030297	265	–	12.19	–	Chitinase 1
Unigene0081457	507	–	12.49	–	Chitinase 5-like isoform 1
Unigene0030294	210	–	12.62	–	Chitinase A
Unigene0080172	554	–	12.77	–	Chitinase 1
Unigene0029489	231	–	13.79	–	Chitinase
Unigene0043619	217	12.57	15.22	15.55	Chitinase 7
Unigene0043616	307	16.40	18.17	18.24	Chitinase
Unigene0002853	249	12.56	–	–	Class IV chitinase precursor
Unigene0011732	256	12.33	–	–	Chitinase
Unigene0043618	225	12.71	–	–	Chitinase
Unigene0119139	1221	–	−3.48	−11.66	Peroxidase 4
Unigene0117425	1206	6.11	5.01	5.08	Peroxidase 73
Unigene0026142	1956	3.16	5.36	4.95	Glutathione peroxidase
Unigene0030009	1082	–	5.85	4.10	Peroxidase 15-like
Unigene0020236	1209	4.25	6.72	6.62	Peroxidase precursor, partial
Unigene0028092	1231	5.65	8.37	8.18	Peroxidase 17-like
Unigene0040032	1473	11.45	9.71	11.61	Glutathione peroxidase
Unigene0082432	591	11.12	11.03	11.74	Ascorbate peroxidase
Unigene0004739	641	–	11.10	11.53	Catalase-peroxidase
Unigene0089597	388	–	11.53	–	Glutathione peroxidase
Unigene0013722	305	–	11.64	–	Peroxidase 27-like
Unigene0079731	482	11.61	11.91	–	Peroxidase 4
Unigene0091588	263	–	12.09	–	Peroxidase superfamily protein
Unigene0014855	329	–	12.24	–	Peroxidase/catalase
Unigene0022272	262	–	12.30	12.82	Peroxidase 72
Unigene0061718	1331	–	13.95	14.57	Peroxidase superfamily protein
Unigene0043744	842	13.26	12.69	13.13	Proteinase inhibitor

“*–”indicates that the expression level of the genes after infection with F. mangiferae is not significantly different as relative to that water-inoculated*.

### Differential expression of WRKY transcription factors

WRKY transcription factors form one of the largest protein superfamilies in plants and can regulate various defense processes, as well as play important roles in controlling the transcription of defense-related genes (Pandey and Somssich, 2009; Rushton et al., 2010). KEGG analysis showed that a total of 12 WRKY genes were differentially expressed. Seven, eleven, and five WRKY genes were significantly and differentially expressed at the three time points after *F. mangiferae* inoculation (Additional File 8). Among them, Unigene0024431 (WRKY transcription factor 26), Unigene0075288 (WRKY transcription factor 44-like), Unigene0057353 (WRKY transcription factor 72), and Unigene0010023 (WRKY transcription factor 29) were induced at two time points. The expression level of Unigene0004116 (WRKY transcription factor 27-like) was not significant at 75 days but strongly induced by *F. mangiferae* at 45 days post-infection. These results suggest that the WRKY proteins might function as key positive regulators in mango defense against infection by *F. mangiferae* during colonization.

### Differential expression of phenylpropanoid biosynthesis genes

Phenylalanine ammonia lyases (PALs), sometimes classified as PR proteins, are involved in the biosynthesis of phenolpropanoids, phytoalexins, and monolignols to inhibit pathogens from penetrating cell walls (Hasegawa et al., 2010; Okada, 2011). The gene Unigene0054337, putatively encoding a shikimate O-hydroxycinnamoyltransferase, was suppressed more than 10-fold at 45 and 120 days but not significantly at 75 days after inoculation with *F. mangiferae*. Six chalcone synthases involved in the early steps of flavonoid biosynthesis were upregulated at 75 days (5.65- to 15.23-fold). The putative isoflavone 7-O-methyltransferase Unigene0058326, which presumably methylates 7,4-dihydroxyiso-flavone (daidzein) and 5,7,4-trihydroxyisoflavone (genistein) to yield isoformononetin and prunetin, was suppressed over 2-fold at all three time points. Eight genes encoding PALs were similarly induced by *F. mangiferae* for at least one time point (Additional File 8).

Genes involved in cell-wall modifications were also differentially regulated. Three genes, namely, Unigene0037555, Unigene0013637, and Unigene0078222, putatively encoding cinnamyl-alcohol dehydrogenase, which is responsible for the last enzymatic step in monolignol biosynthesis, were induced over 5-fold at all three time points in response to *F. mangiferae*. By contrast, another member of this gene family, that is, Unigene0012635, was suppressed over 11-fold at 120 days. Furthermore, three putative callose synthases, namely, Unigene0076978, Unigene0064731, and Unigene0128362, were suppressed at all three time points. Several putative pectinesterase genes were differentially expressed; two different genes, Unigene0054305 and Unigene0014015, were induced at all three time points (11.53-, 13.42-, and 13.86-fold; 12.94-, 14.46-, and 14.24-fold, respectively), whereas another gene (Unigene0038718) was suppressed over 10-fold at 75 and 120 days. The gene Unigene0021404, which encodes a pectin methylesterase inhibitor domain-containing protein predicted to prevent or reduce the activity of pectin methylesterase, was suppressed 12.57-, 12.57-, and 2.96-fold at 45, 75, and 120 dpi, correspondingly (Additional File 8).

### Differential expression of signal transduction genes

Hormonal signaling has been reported as a downstream immune response in many studies of pathogen–host interactions (Bari and Jones, 2009). To investigate the transcriptional changes in gene classes involved in *F. mangiferae* perception and signaling, genes with the KEGG pathway associated with plant hormone signal transduction (KO4075) and the GO term associated with the signal transduction process (GO:0007165) were identified. A total of 93 DEGs were found among all datasets by using log_2_-fold ratio ≥5 as a cutoff. Among the genes associated with signal transduction, 58 were identified from the pathway of plant hormone signal transduction (KO4075; Additional File 9).

The gene Unigene0010084, putatively encoding a lipoxygenase isoform 3, which is involved in the biosynthesis of JA, was induced more than 10-fold in response to *F. mangiferae* at all three time points. The ET-responsive transcription factors (ERFs) and 1-aminocyclopropane-1-carboxylate oxidases (ACOs) are known to be involved in the last step of ET biosynthesis (Fujimoto et al., 2000) and play crucial roles in regulating plant responses to pathogen inoculation (Berrocal-Lobo et al., 2002; Huang et al., 2004). At 75 days post-inoculation, the genes Unigene0079845 and Unigene0035078, putatively encoding ACO genes, were upregulated by 13.08- and 13.00-fold in response to *F. mangiferae*; by contrast, two other members of this gene family, Unigene0038132 and Unigene0123919, were suppressed by 11.66- and 5.08-fold, respectively. Moreover, two putative ACO genes (Unigene0079599 and Unigene0092709) were induced more than 10-fold at 45 days but not significantly at 75 and 120 days in response to *F. mangiferae*. Six putative ERF genes (Unigene0075508, Unigene0033494, Unigene0025794, Unigene0013858, Unigene0079510, and Unigene0033493) showed over 5-fold induction at all three time points. Another ERF gene (Unigene0051908) was suppressed 10.72-fold at 75 days only. The induction of ERFs and ACOs suggests that ET was involved in the resistance against *F. mangiferae*. Two putative auxin-induced SAUR family genes (Unigene0037759 and Unigene0039593), known to be rapidly and transiently upregulated in response to auxin (McClure and Guilfoyle, 1987; Markakis et al., 2013), were suppressed at 75 and 120 days but constitutively expressed at 45 days. Two other putative auxin-induced SAUR family genes (Unigene0021557 and Unigene0012223) were induced in response to *F. mangiferae* (Additional Files 8, 9).

### Differential expression of protein kinase genes

Plants can recognize potential microbial pathogens through PAMPs by host sensors. Most of these plant receptors belong to the receptor-like kinase (RLK) family (Liu et al., 2009; Beck et al., 2012). In our study, 275 protein kinases were found to be differentially expressed in mango buds after *F. mangiferae* inoculation (Additional File 8). Five, 21, 20, 13, 47, 42, and 53 DEGs were identified encoding calcium-dependent protein kinases, cysteine-rich receptor-like protein kinases, G-type lectin S-receptor-like serine threonine-protein kinases, LRR protein kinases, LRR receptor-like serine/threonine-protein kinases, serine/threonine protein kinases, and proline-rich receptor-like protein kinases, correspondingly (Additional File 8). Most calcium-dependent protein kinase, G-type lectin S-receptor-like serine/threonine-protein kinase, and proline-rich receptor-like protein kinase genes were upregulated, whereas a majority of LRR protein kinase, LRR receptor-like serine/threonine-protein kinase, and serine/threonine protein kinase genes were downregulated. Interestingly, many genes encoding LRR receptor-like serine/threonine protein kinases were suppressed after infection. This finding indicates that *F. mangiferae* secretes and delivers effectors into the mango bud cells during infection to suppress immune signaling, leading to MMD. This result agrees with the transcriptomic analysis of the *Phytophthora pisi*–pea interaction, in which subsets of pathogen effectors and host receptor genes are induced and repressed, respectively (Hosseini et al., 2015). Furthermore, the data indicate that some LRR receptor-like serine/threonine protein kinase-encoding genes in mango are specifically activated at each time point after inoculation with *F. mangiferae*. Mitogen-activated protein kinase (MAPK) and MAPK kinase (MAPKK) genes have been characterized in the response of plants to fungal infection (Rodriguez et al., 2010; Kishi-Kaboshi et al., 2012). Consistent with this widely accepted model, we found two MAPK, one MAPKK, and four MAPKK kinase (MAPKKK) genes involved in the response of mango buds to *F. mangiferae* infection. DEGs encoding MAPK were downregulated, and this finding suggests that MAPKs act positively and negatively in mango resistance to *F. mangiferae*, but the exact roles of MAPK still require further research.

Overall, our findings indicating that many defense-related genes including PR genes, WRKY transcription factors, protein kinases, phenylpropanoid, and signal transduction genes are upregulated after *F. mangiferae* inoculation, suggest that these genes play essential roles in MMD resistance in mango.

## Author contributions

Conceived and designed the experiments: FL, JW, RZ, Performed the experiments: FL, JW, Analyzed the data: XO, Wrote the paper: FL, Contributed reagents/materials/analysis tools: RZ.

### Conflict of interest statement

The authors declare that the research was conducted in the absence of any commercial or financial relationships that could be construed as a potential conflict of interest.

## Supplementary material

Additional File 1 **Number of unigenes matching the species using BLASTx in the Nr database**.

Additional File 2 *****Mangifera***_***indica***-Unigene.fa.cog.gene.annot**.

Additional File 3 **KEGG pathway annotation for transcript sequence in mango**.

Additional File 4 **DEGs**.

Additional File 5 **Gene ontology (GO) analysis of DEGs**.

Additional File 6 **Significantly enriched KEGG pathways of DEGs by ***F. mangiferae*** infection**.

Additional File 7 **Eleven genes selected for validation of expression level with qPCR**.

Additional File 8 **Identification and Functional categorization of ***F. mangiferae***-responsive genes**.

Additional File 9 **DEGs in the common enriched KEGG pathway “Plant hormone signal” (ko04075)**.

Figure S1 **Frequency distribution of Contig lengths in Illumina HiSeq™ 2000 sequencing data**.

Figure S2 **Frequency distribution of Unigene lengths in Illumina HiSeq™ 2000 sequencing data**.

Figure S3 **Statistical comparison 4 database gene Venn diagram**.

Figure S4 **COG annotations of putative proteins in mango**.

Table S1 **COG annotations of putative proteins in mango bud following inoculation with ***Fusarium mangiferae*****.
