# Supplementary figures and images for: Transcription Profiling Analysis of Mango–Fusarium mangiferae Interaction
Source: Front Microbiol. 2016 Sep 14;7:1443. doi: 10.3389/fmicb.2016.01443 (PMC5022174; doi:10.3389/fmicb.2016.01443)

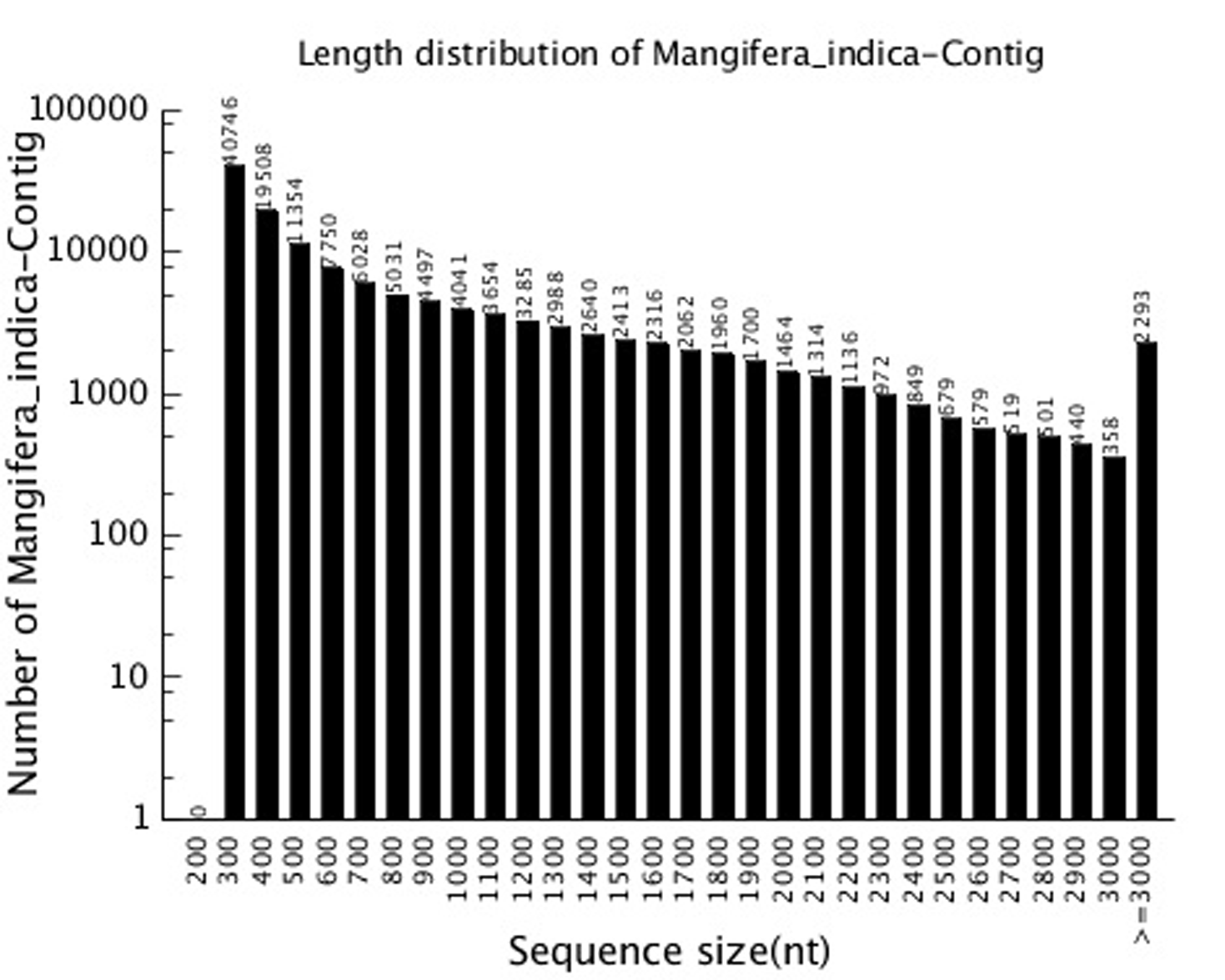

Supplement: Figure S1 — Frequency distribution of Contig lengths in Illumina HiSeq™ 2000 sequencing data. [file Image1.TIF]

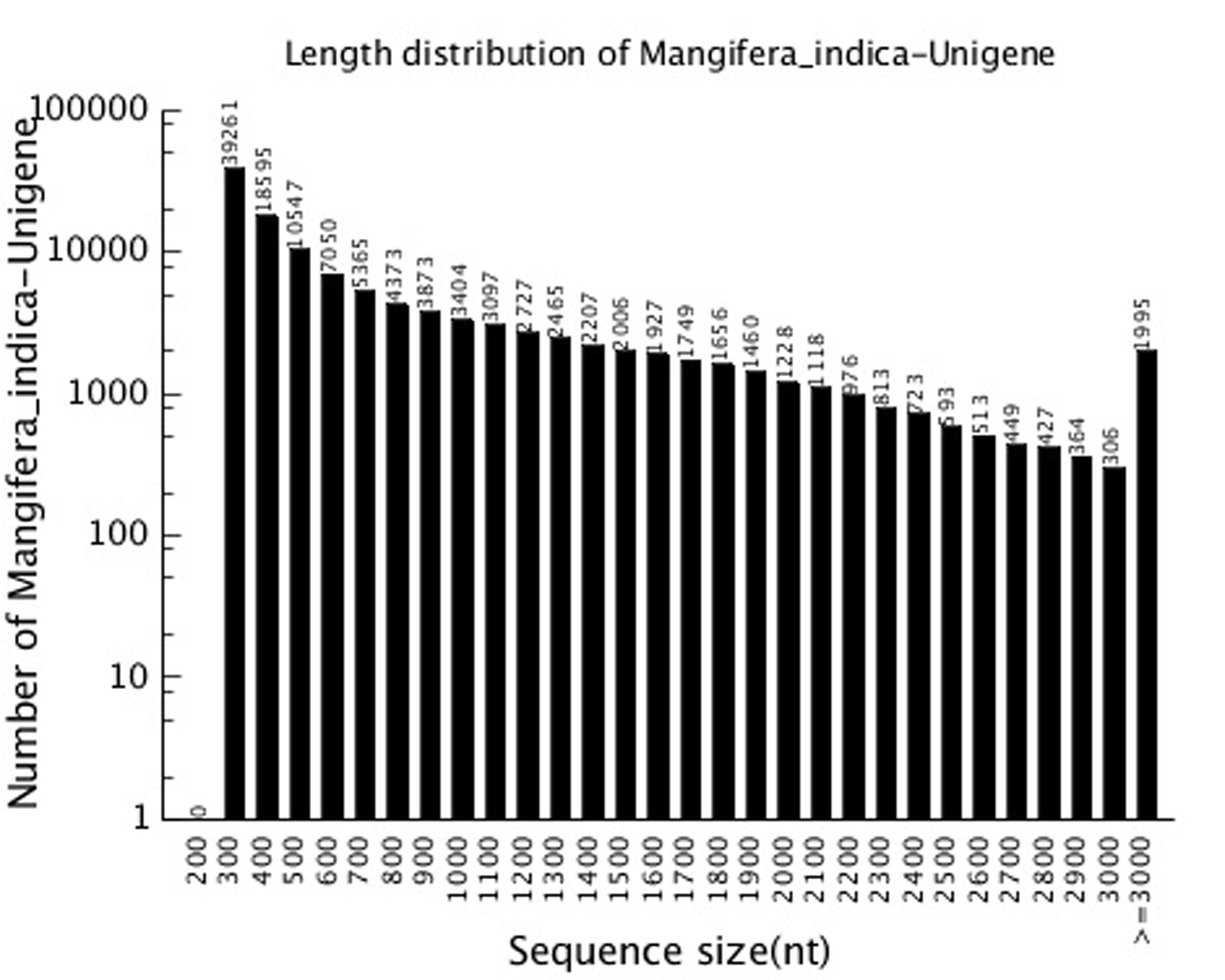

Supplement: Figure S2 — Frequency distribution of Unigene lengths in Illumina HiSeq™ 2000 sequencing data. [file Image2.TIF]

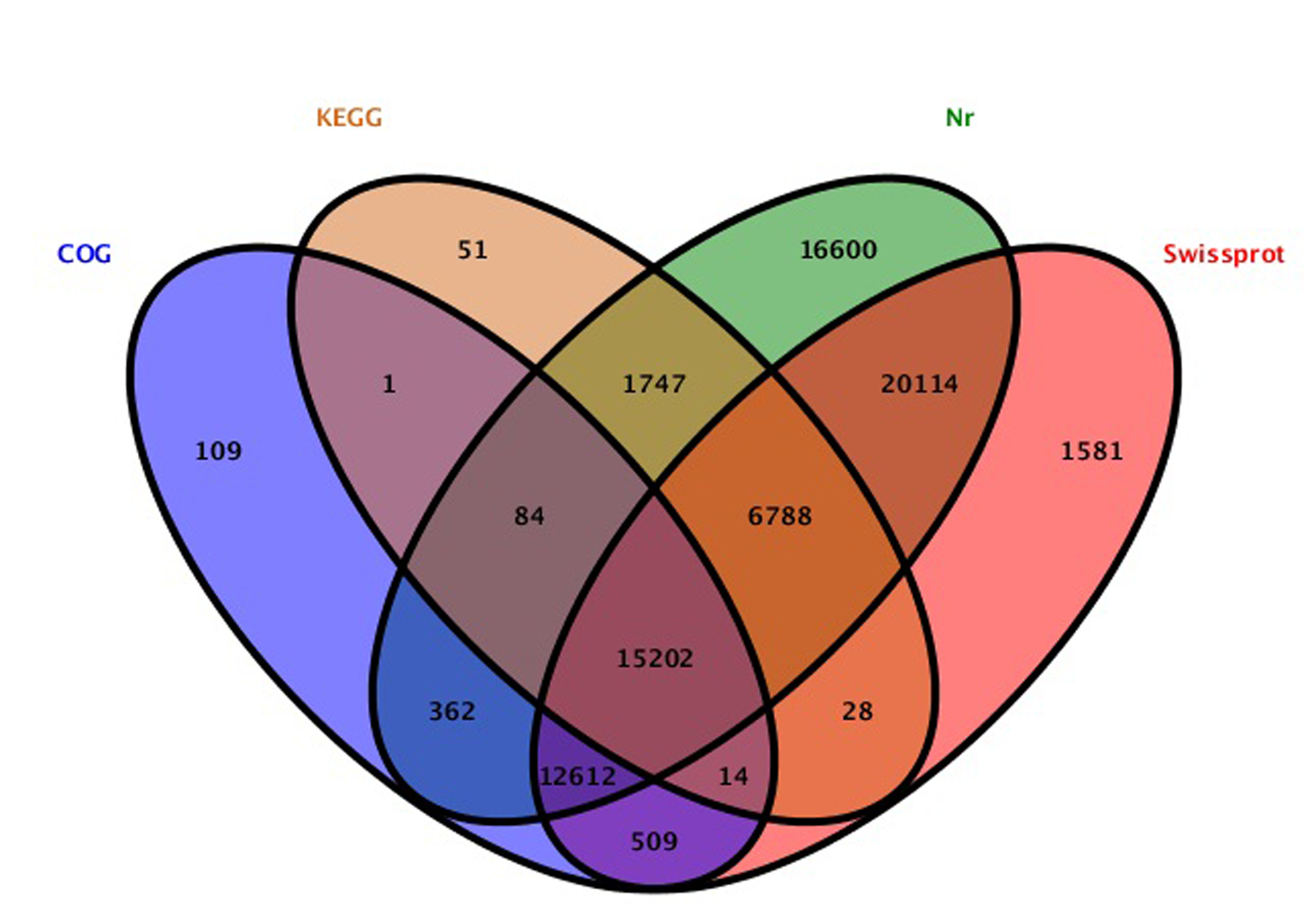

Supplement: Figure S3 — Statistical comparison 4 database gene Venn diagram. [file Image3.TIF]

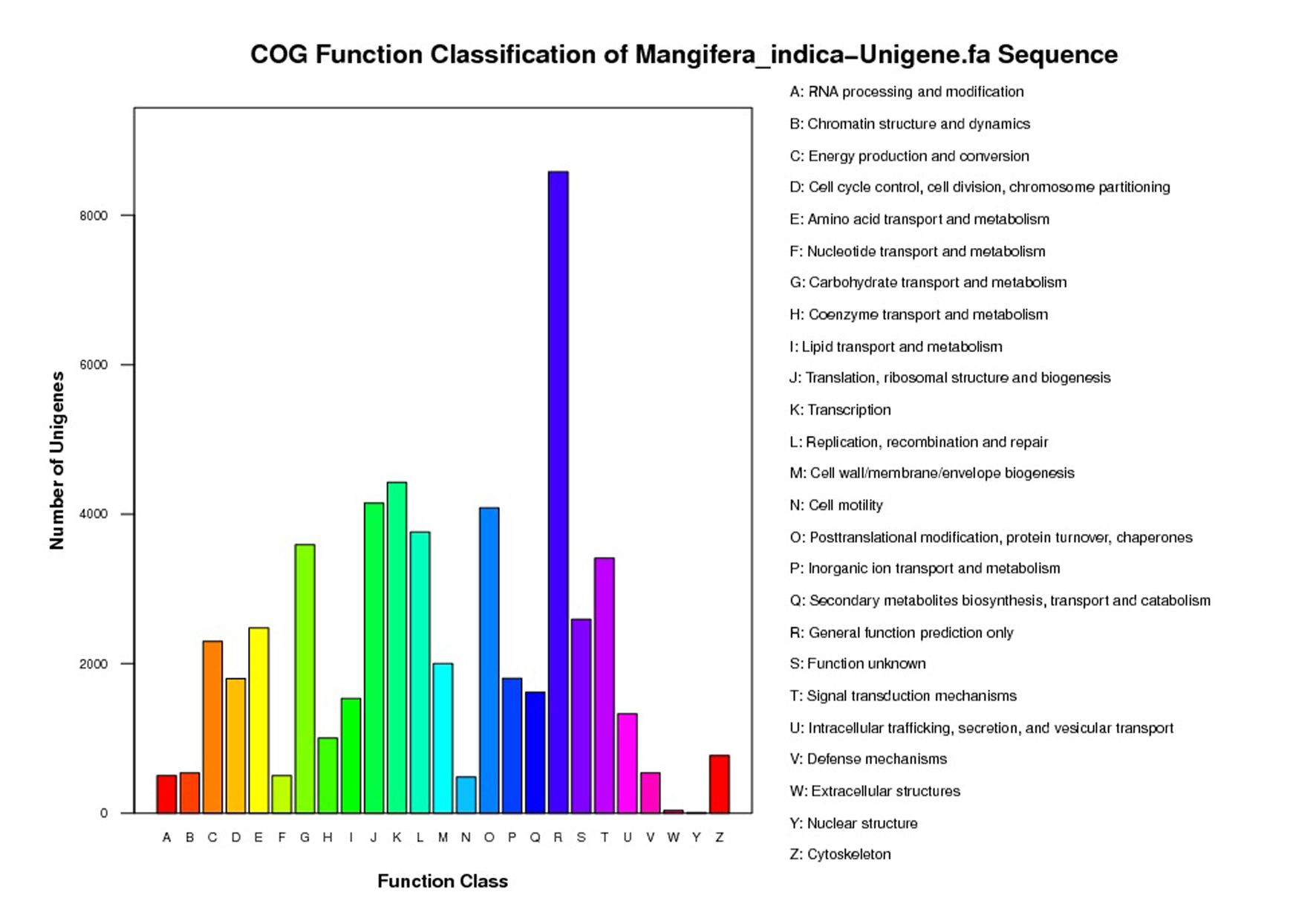

Supplement: Figure S4 — COG annotations of putative proteins in mango. [file Image4.TIF]
