# Supplementary material for: Transcription Profiling Analysis of Mango–Fusarium mangiferae Interaction
Source: Front Microbiol. 2016 Sep 14;7:1443. doi: 10.3389/fmicb.2016.01443 (PMC5022174; doi:10.3389/fmicb.2016.01443)
Supplement: Table S1 — COG annotations of putative proteins in mango bud following inoculation with Fusarium mangiferae. [file Table1.DOC]

Table S1 COG annotations of putative proteins in mango bud following inoculation with *Fusarium mangiferae*

| Functional-Categories | Gene-Number | Percentage |
| --- | --- | --- |
| General function prediction only | 8578 | 15.94% |
| Transcription | 4428 | 8.23% |
| Translation, ribosomal structure and biogenesis | 4148 | 7.71% |
| Posttranslational modification, protein turnover, chaperones | 4085 | 7.59% |
| Replication, recombination and repair | 3760 | 6.99% |
| Carbohydrate transport and metabolism | 3589 | 6.67% |
| Signal transduction mechanisms | 3409 | 6.33% |
| Function unknown | 2592 | 4.82% |
| Amino acid transport and metabolism | 2476 | 4.60% |
| Energy production and conversion | 2299 | 4.27% |
| Cell wall/membrane/envelope biogenesis | 2002 | 3.72% |
| Inorganic ion transport and metabolism | 1803 | 3.35% |
| Cell cycle control, cell division, chromosome partitioning | 1801 | 3.35% |
| Secondary metabolites biosynthesis, transport and catabolism | 1617 | 3.00% |
| Lipid transport and metabolism | 1534 | 2.85% |
| Intracellular trafficking, secretion, and vesicular transport | 1326 | 2.46% |
| Coenzyme transport and metabolism | 1002 | 1.86% |
| Cytoskeleton | 770 | 1.43% |
| Defense mechanisms | 537 | 1.00% |
| Chromatin structure and dynamics | 535 | 0.99% |
| RNA processing and modification | 499 | 0.93% |
| Nucleotide transport and metabolism | 499 | 0.93% |
| Cell motility | 482 | 0.90% |
| Extracellular structures | 36 | 0.07% |
| Nuclear structure | 9 | 0.02% |
